# Prevalence, Location, and Variations of the Posterior Superior Alveolar Canal With Age and Gender in the Indian Population: A Cone Beam Computed Tomography (CBCT)-Based Retrospective Study

**DOI:** 10.7759/cureus.60658

**Published:** 2024-05-20

**Authors:** Kamalkishor Mankar, Humaira Siddique, Abhay Kolte, Adiba Siddique, Vaishnavi Mishra, Anjali Borkar

**Affiliations:** 1 Periodontics and Implantology, Ranjeet Deshmukh Dental College and Research Centre, Nagpur, IND; 2 Dentistry, Datta Meghe Medical College, Datta Meghe Institute of Higher Education and Research, Nagpur, IND; 3 Microbiology, Jawaharlal Nehru Medical College, Datta Meghe Institute of Higher Education and Research, Wardha, IND; 4 Anaesthesiology, Datta Meghe Medical College, Datta Meghe Institute of Higher Education and Research, Nagpur, IND

**Keywords:** sinus floor elevation, ms, lateral sinus wall, cone beam computed tomography, posterior superior alveolar artery canal

## Abstract

Background: Enhancing the availability of bone in the vertical dimension for implant insertion is thought to be possible through implant site preparation using direct or indirect sinus lift. The posterior superior alveolar (PSA) canal is extremely vulnerable to trauma during this procedure. The anatomy of this region should be thoroughly evaluated to prevent traumatizing this artery and eventual perioperative bleeding. Due to a lack of relevant knowledge and the clinical importance of this problem, the position, diameter, detectability, and proximity of this canal to the alveolar ridge were assessed on cone beam computed tomography (CBCT) scans which were the main objectives of this study.

Methodology: A total of 240 CBCT scans were examined, and the position of the PSA canal, its diameter, the perpendicular distance from the inferior border of the PSA canal to the alveolar crest, and the perpendicular distance from the inferior border of the canal to the maxillary sinus floor was measured.

Results: Intraosseous PSA canals were the most prevalent, followed by intrasinusal and extraosseous canals. Males had larger canal diameters and greater distances between the maxillary sinus floor and alveolar crest and the canal (*P *< 0.05).

Conclusion: CBCT was proven to be a useful method for assessing and localizing the PSA artery to prevent intraoperative bleeding and further complications.

## Introduction

Implants in dentistry have proven to be the most popular form of oral rehabilitation therapy in the modern era. For dental implant placement, adequate bone volume is a crucial prerequisite. Implant insertion via conventional procedures may not be the best option in regions like the maxillary posteriors, in which bone atrophy, as well as pneumatization occurring in the maxillary sinus (MS), are frequent consequences of tooth loss. To increase the vertical dimensions of bone for dental implant placement, sinus lift surgery has shown to be a highly reliable surgical treatment for implant site preparation [1].

However, during this surgery, the posterior superior alveolar (PSA) canal and the artery are extremely prone to damage [2,3]. To avoid perioperative or postoperative issues that could cause hemorrhage, it is crucial to have a complete grasp of the anatomy of MS and its related structures before performing a sinus lift treatment [4]. Because only a minor artery is injured, intraoperative bleeding might not be a life-threatening problem, but it might make surgery more challenging in some situations [5]. Before commencing any surgical procedure, it is essential to pinpoint the accurate location and diameter of the posterior superior alveolar canal (PSAC) to prevent traumatizing this artery. An increased risk of injury is linked to age-related MS enlargement, alveolar crest resorption, and other advanced surgical procedures performed in this region [6].

Cone-beam computed tomography (CBCT) was initially applied in the 90s. It is a kind of digital imaging technique that accurately identifies anatomical landmarks like the PSAC as well as the dimensions of bone [7]. The main advantages of CBCT as compared to computed tomography (CT) are lower patient radiation, dosage, and reduced cost [8]. Because of the importance of the topic in clinical practice, the paucity of sufficient data in this area, and the findings of earlier research are controversial regarding the precise location of the PSAC, this study was carried out to evaluate the prevalence, diameter, location, and relationship of the PSAC with the alveolar ridge and floor of the sinus on CBCT images.

## Materials and methods

The study was carried out at the Institute's Periodontics and Implant Dentistry department from March 2022 to August 2022. The protocol of the study had been accepted by the Institutional Ethics Committee (EC/NEW/INST/2020/687) and adhered to the 2013 revision of the 1975 Helsinki Declaration. Patients aged between 20 and 70 years who had a total of 120 CBCT scans, or 240 maxillary sinuses (MSs), were chosen and evaluated. These individuals underwent CBCT imaging for dental treatment, mostly for dental implant treatment planning, intrabody defects, and impacted third molar extractions. The study included high-quality CBCT images. The images were excluded if there was a fracture in the upper jaw or any pathological lesions involving the upper jaw. All images were analyzed by two examiners. For assessing the intra-examiner variance, each examiner measured randomly selected 50 CBCT images twice at an interval of at least one month.

Radiographic image analysis

**Figure 1:**
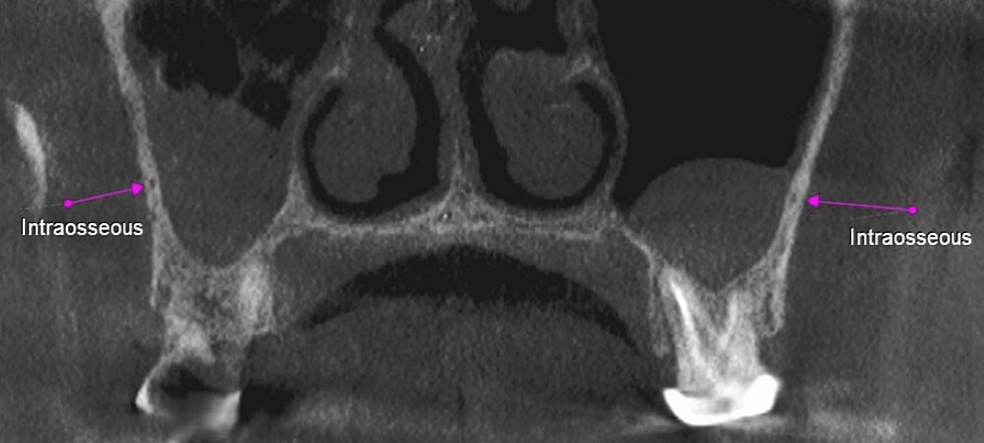
Position of the posterior superior alveolar artery canal (pink arrow).

**Figure 2:**
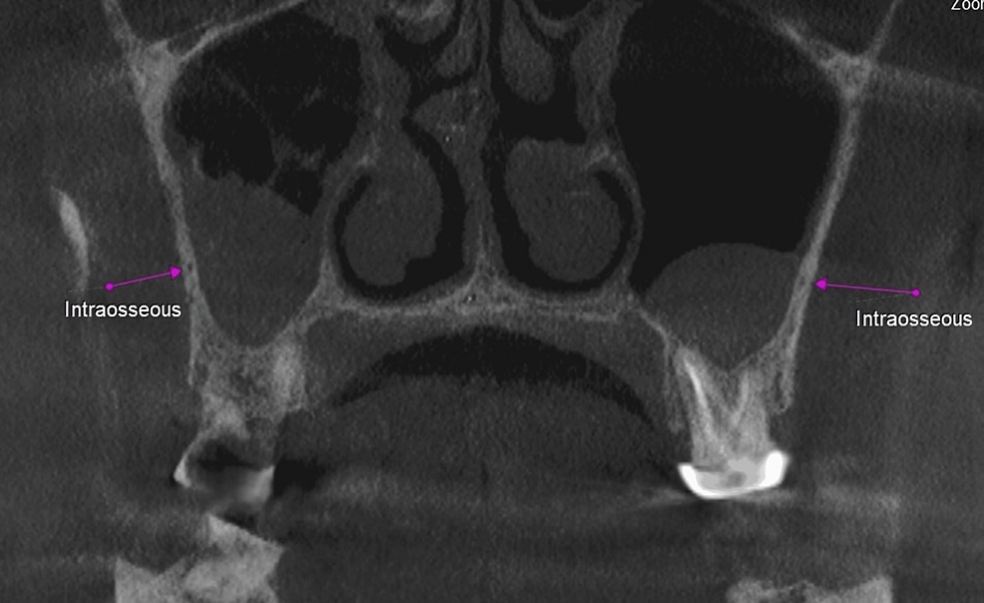
Pink arrows showing the intraosseous posterior superior alveolar canal (PSAC).

**Figure 3:**
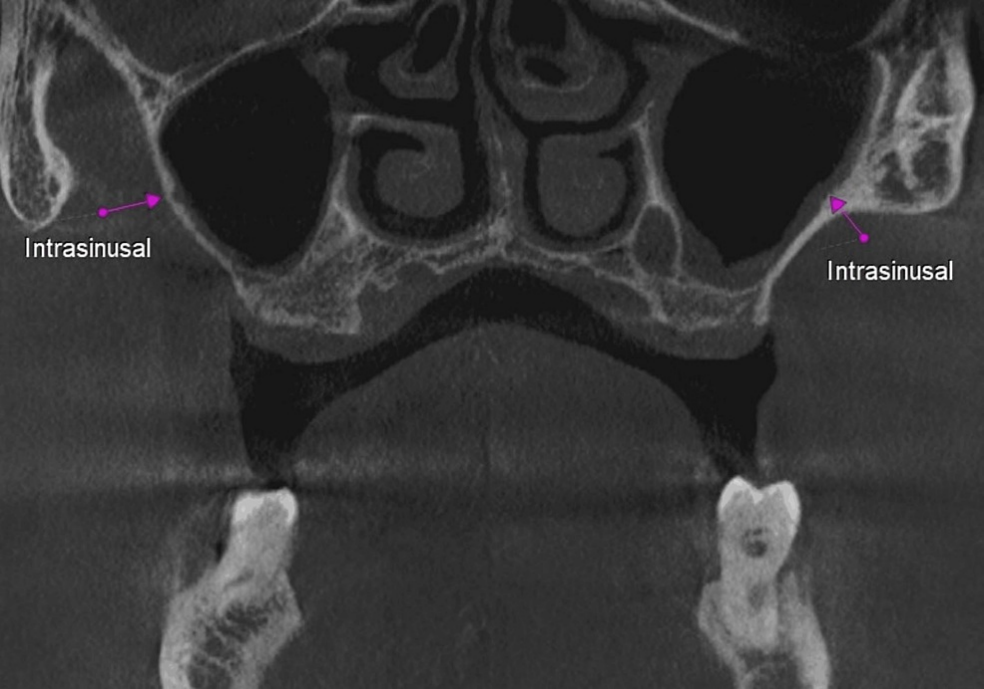
Pink arrow showing the intrasinusal posterior superior alveolar canal (PSAC).

**Figure 4:**
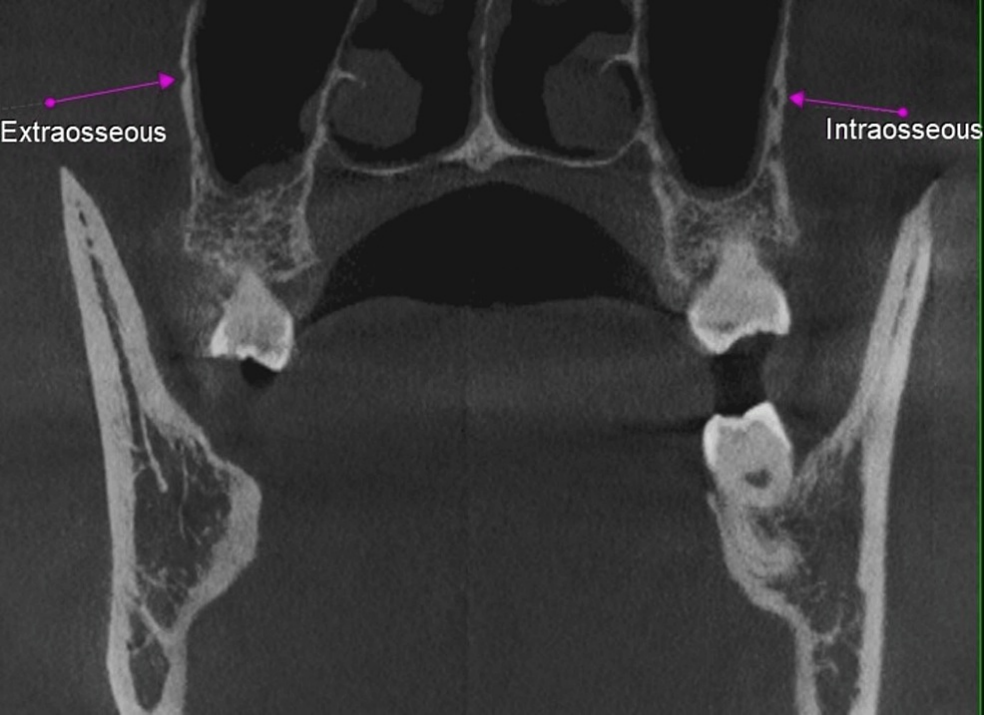
Pink arrow showing the extraosseous posterior superior alveolar canal (PSAC) on the left side and the intraosseous PSAC on the right side.

**Figure 5:**
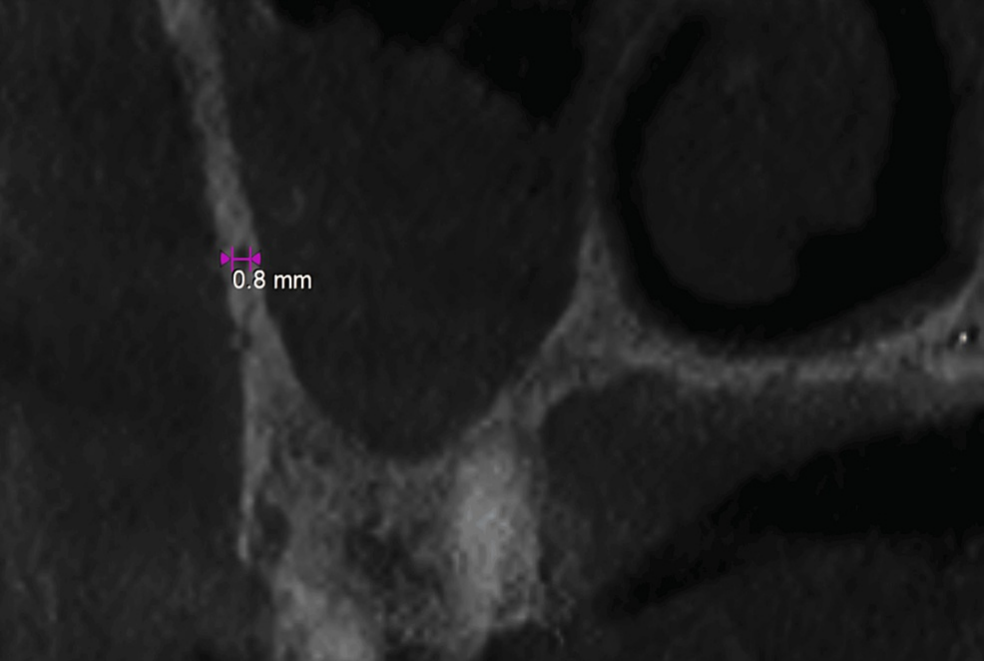
Diameter of the posterior superior alveolar artery canal.

At 84 kVp and 16 mA, Orthophos® XG 3D/Ceph, a product of Sirona Dental Systems GmbH, Germany, was used to obtain all CBCT pictures. Commercially accessible CBCT software was used to analyze the data. The coronal section of the CBCT image was reconstructed to determine the position of the PSAC concerning three locations: intraosseous, intrasinusal, and extraosseous (Figures 1-5).

The diameter of the PSAC concerning three types: 1) diameter smaller than 1 mm, 2) diameter in a range of 1-2 mm, and 3) diameter more than 2 mm (Figure 5).

**Figure 6:**
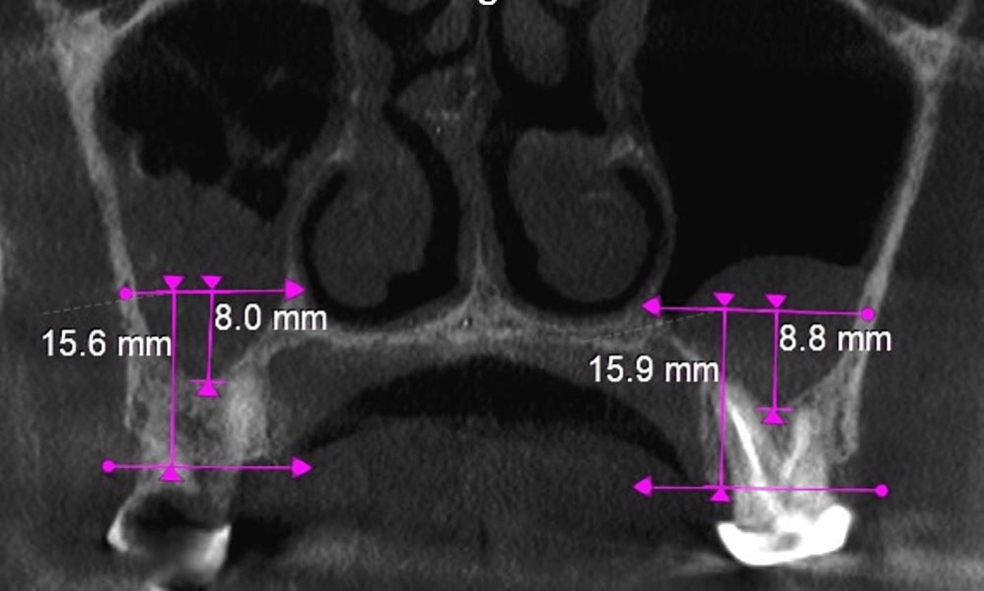
Distance from the inferior border of the PSA artery canal to the alveolar crest and distance from the inferior border of the PSA artery canal to the sinus floor. The right maxillary sinus showing 8 mm distance from the PSAC to the floor of sinus and 15.6 mm distance from PSAC to the alveolar crest. Similarly, left maxillary sinus showing 8.8 mm distance from the PSAC to the floor of the sinus and 15.9 mm distance from PSAC to the alveolar crest. PSA, posterior superior alveolar; PSAC, posterior superior alveolar canal

The distance measured was a perpendicular distance from the inferior margin of the PSAC to the crest of the alveolar bone. Similarly, the distance measured was a perpendicular distance from the inferior margin of the PSAC to the MS. Right MS showing 8 mm distance from the PSAC to the floor of sinus and 15.6 mm distance from PSAC to the alveolar crest. Similarly left MS showing 8.8 mm distance from the PSAC to the floor of sinus and 15.9 mm distance from PSAC to the alveolar crest (Figure 6).

All the measurements were done using the measurement tools of the CBCT software. Microsoft Office Excel 2010 was used to enter the data, and IBM SPSS Statistics for Windows, Version 20.0 (IBM Corp., Armonk, NY) was used to analyze the findings. For quantitative data, descriptive statistics like mean and standard deviation were generated. The Shapiro-Wilk test was used to evaluate the data. To compare research variables between the male and female populations, an unpaired t-test was performed.

## Results

The total age of the patients ranged from 20 to 70 years old, with a mean of 45.6±15.26 years. Overall, 120 CBCT scans (240 MSs) were evaluated, with an equal distribution of 60 males and 60 females. Approximately 78% of CBCT scans showed evidence of the PSAC. The diameter of the PSAC was measured on both the right and left sides of the maxilla in males and females. Although there were more detections of the PSAC on the right side, this difference was not statistically significant (*P* > 0.05).

Extraosseous PSACs were more prevalent on the right side, while intraosseous PSACs were more common on the left. Table 1 shows the distance from the PSAC to the alveolar crest ridge in both genders. The mean distance from the crest of the alveolar bone at extraosseous levels on the right side was 21.33 ± 3.8 mm in males and 21.33 ± 3.8 mm in females, while on the left side, it was 17.27 ± 2.16 mm in males and 17.33 ± 2.03 mm in females. No statistically significant difference (*P *> 0.05) between males and females was observed.

**Table 1:** Comparative analysis of posterior superior alveolar canal characteristics by gender in the Indian population. All distances and diameters are measured in millimeters (mm). Statistical significance is noted by *P*-values, with significant findings indicated by *P *< 0.05. PSAC, posterior superior alveolar canal; extraosseous, outside the bones; intraosseous, inside the bones; intrasinusal, within the sinus; MS, maxillary sinus; SD, standard deviation; mean ± SD, average measurement plus or minus the standard deviation

Feature	Location	Male (mean ± SD)	Female (mean ± SD)	*t*-value	*P*-value
Diameter - Right-side PSAC	Extraosseous	1.03 ± 0.48	0.84 ± 0.21	0.0	0.267
	Intraosseous	0.97 ± 0.34	1.04 ± 0.45	-0.116	0.450
	Intrasinusal	1.03 ± 0.39	0.90 ± 0.13	1.148	0.340
Diameter - Left-side PSAC	Extraosseous	1.06 ± 0.37	1.07 ± 0.4	1.148	0.908
	Intraosseous	1.03 ± 0.48	0.84 ± 0.21	-0.759	0.267
	Intrasinusal	0.96 ± 0.1	0.96 ± 0.1	0.981	1.000
Distance to alveolar crest - right	Extraosseous	21.33 ± 3.8	21.33 ± 3.8	0.0	1.000
	Intraosseous	17.25 ± 1.94	17.28 ± 1.97	-0.061	0.951
	Intrasinusal	16.33 ± 3.76	16.28 ± 3.55	0.032	0.975
Distance to alveolar crest - left	Extraosseous	16.33 ± 3.76	16.28 ± 3.55	0.032	0.975
	Intraosseous	17.27 ± 2.16	17.33 ± 2.03	-0.139	0.890
	Intrasinusal	17.17 ± 2.05	17.18 ± 2.04	-0.011	0.991
Distance to MS floor - right	Extraosseous	10.2 ± 2.25	10.2 ± 2.25	0.0	1.000
	Intraosseous	8.21 ± 1.9	8.23 ± 1.94	-0.057	0.955
	Intrasinusal	8.72 ± 1.62	8.5 ± 1.68	0.291	0.774
Distance to MS floor - left	Extraosseous	8.72 ± 1.62	8.5 ± 1.68	0.291	0.774
	Intraosseous	8.15 ± 1.83	8.17 ± 1.84	-0.036	0.971
	Intrasinusal	8.3 ± 1.73	8.32 ± 1.7	-0.026	0.979

## Discussion

According to our analysis, nearly all of the CBCT images evaluated for the study showed the presence of the PSAC. This results in a somewhat greater prevalence than the 92% reported by Fayek et al. [1], the 89.3% reported by Ilgüy et al. [6], the 87% reported by Tehranchi et al. [9], the 82% reported by Apostolakis and Bissoon [10], and the 94% reported by Anamali et al. [11]. These variations may result from variations in the demographics and research methodology used in various studies. Furthermore, a large number of studies revealed lower prevalence rates. For example, in investigations that used CT, 52.9% was reported by Elian et al. [12], 55% by Mardinger et al. [4], 52% by Kim et al. [13], and 64% by Güncü et al. [7]. These differences can be ascribed to CBCT's superior spatial resolution over conventional CT imaging, which aids in the detection of tiny canals. Additionally, the capacity of the observer to identify the canal may be further limited because, in several earlier studies, such as the one by Mardinger et al. [4], the identification of the canal was made based on images formed on film.

The intraosseous position accounted for 75% of all canal positions. This prevalence rate (68.2% and 71.1%, respectively) is nearly identical to the values published by Ilguy et al. [6] and Güncü et al. [7]. No previous research has examined the diameter of the left and right PSACs, making this study unique in that regard. The results of this investigation are displayed. According to the study's findings, the PSAC diameter was essentially the same in the right and left MSs. Therefore, it would be simple to detect the diameter of the opposite side when interpreting the canals in CBCT scans, saving time both during interpretation and when preparing for implant placement scenarios.

According to research by Khojastehpour et al. [14], the distance between the PSAC and the alveolar crest was found to be almost the same in both males and females. The current investigation similarly reported that the distance between the PSAC and the alveolar crest was almost identical in both males and females, as demonstrated.

In this study, the mean distance between the floor of MS and the inferior margin of the PSA artery canal was found to be somewhat greater in males than in females. These results coincided with those of Güncü et al. [7] and Mardinger et al. [4]. This discrepancy may result from anatomical differences in the locations of the arteries; also, it has been noted that males have a larger MS capacity than females.

The majority of research on the location of the PSACs has been conducted on dry skulls with finite information about the patient’s age, sex, or ethnicity. Ang et al. confirmed the different locations of the PSAC in relation to the lateral wall of the MS with no gender influence [15]. This study used CBCT that provided additional information on age, gender, and ethnicity. Since CBCT is gaining increased popularity, images are more readily available and a larger sample can be studied. Furthermore, it provides subjects with specific, predetermined characteristics and allows precise measurements. Since it is crucial to find the most precise position of the PSAC for several maxillary surgical procedures, the observations made in this research will be beneficial to clinicians. Therefore, it is quite clear that a combination of the aforementioned measurements can help to trace the PSAC accurately. However, further research is needed to substantiate these findings, and future studies involving a larger sample are required.

Limitations

Limitations of the study include limited sample size and the inability to stratify analysis into different age groups of patients. The authors recommend that more research be done on larger samples. Stratification of subjects into different ethnic groups can be useful to determine if there are genetic differences between ethnicities.

## Conclusions

The study reveals that intraosseous and intrasinusal canals are the most common in the PSA canals, making understanding their variations and locations crucial for surgical procedures. CBCT is proposed as a valuable tool for this purpose, as it provides detailed 3D images of the maxillofacial region, allowing clinicians to accurately determine the location and diameter of the PSA artery. This information aids in surgical planning, allowing practitioners to anticipate potential challenges and take appropriate precautions during procedures in the posterior maxilla, ultimately contributing to safer and more effective clinical outcomes in dental and maxillofacial surgery.
